# Investigation of Surface Properties and Free Volumes of Chitosan-Based Buccal Mucoadhesive Drug Delivery Films Containing Ascorbic Acid

**DOI:** 10.3390/pharmaceutics14020345

**Published:** 2022-02-01

**Authors:** Katalin Kristó, Szilvia Módra, Viktória Hornok, Károly Süvegh, Krisztina Ludasi, Zoltán Aigner, András Kelemen, Tamás Sovány, Klára Pintye-Hódi, Géza Regdon

**Affiliations:** 1 Institute of Pharmaceutical Technology and Regulatory Affairs, University of Szeged, H-6720 Szeged, Hungary; modra.szilvia@gmail.com (S.M.); ludasi.krisztina@szte.hu (K.L.); sovany.tamas@szte.hu (T.S.); hodiklara@szte.hu (K.P.-H.); geza.regdon@pharm.u-szeged.hu (G.R.J.); 2 Department of Physical Chemistry and Materials Science, University of Szeged, H-6720 Szeged, Hungary; vhornok@chem.u-szeged.hu; 3 Department of Nuclear Chemistry, Eötvös Loránd University, H-1518 Budapest, Hungary; suveghk@chem.elte.hu; 4 Department of Applied Informatics, University of Szeged, H-6725 Szeged, Hungary; kelemen.andras.felix@szte.hu

**Keywords:** buccal mucoadhesive film, atomic force microscopy, free volume, mucoadhesive force, structure analysis

## Abstract

Nowadays, the buccal administration of mucoadhesive films is very promising. Our aim was to prepare ascorbic acid-containing chitosan films to study the properties and structures important for applicability and optimize the composition. During the formulation of mucoadhesive films, chitosan as the polymer basis of the film was used. Ascorbic acid, which provided the acidic pH, was used in different concentrations (2–5%). The films were formulated by the solvent casting method. The properties of films important for applicability were investigated, such as physical parameters, mucoadhesive force, surface free energy, and breaking strength. The fine structure of the films was analyzed by atomic force microscopy, and the free volume was analyzed by PALS, which can be important for drug release kinetics and the location of the drug in the film. The applicability of the optimized composition was also tested with two different types of active ingredients. The structure of the films was also analyzed by XRPD and FTIR. Ascorbic acid can be used well in chitosan films, where it can function as a permeation enhancer when reacting to chitosan, it is biodegradable, and can be applied in 2% of our studies.

## 1. Introduction

Nowadays, bioadhesive formulations are increasingly becoming a very important class of dosage in the forms of films [1,2,3], tablets [4], nanoparticles [5,6], etc. Buccal mucoadhesive films are one of the most innovative and promising groups of dosage forms. The advantage of mucoadhesive forms is that the active ingredient can continuously absorb the biological barrier by adhering to the mucosa. Within this, the advantage of buccal films is that they do not have to be swallowed, so they can be used by patients who have difficulty swallowing, and the first-pass effect of the liver can be avoided, which means that a smaller amount of active ingredient is sufficient to reduce side effects. From mucoadhesive polymers, mucoadhesive films that can adhere to the buccal mucosa can be easily produced with simple pharmaceutical technology solutions. A number of polymers that are suitable for this purpose have been used in the literature, such as cellulose derivatives [7], sodium alginate [8], or chitosan. Due to its exceptional biological nature, chitosan polysaccharide (CS) is often used in pharmaceutical and biomedical formulations [9]. Chitosan is a safe, biocompatible, and biodegradable polymer. Chitosan is a linear copolymer composed of randomly distributed β (1 → 4) with N-acetyl glucosamine and glucosamine units. Chitosan is an alkaline substance, and its pKa value is approximately 6.5 [9,10]. The advantage of chitosan is that it is biodegradable, has adequate mucoadhesive properties, is an excellent film-forming polymer, and has permeation enhancer properties [11,12,13]. Some research groups have already studied its applicability in buccal films. Ashri et al. used tenoxicam in a buccal film to treat chronic periodontitis. A film was prepared that released the active ingredient continuously for 6 h and contained less than the dose administered orally [14]. Kolan et al. formulated the sustained release of an ondansetron hydrochloride chitosan film and found it promising in further clinical trials based on studies in rabbits [15]. Chitosan is soluble in acidic environments, so Abouhussein et al. studied how different types of acids affect the physicochemical structure of polymer films. Films made with acetic acid have been found to be more advantageous in terms of oral pH, while films made with lactic acid are more advantageous in terms of flexibility and mucoadhesion [16]. Kumria and co-workers formulated a zitmitriptan-containing chitosan film for the treatment of migraines and optimized its composition by factorial experimental design [17].

One of the important studies in terms of applicability is the study of breaking strength and deformation processes. This is because the elasticity of the film can be inferred from the shape of the deformation curve, which can be influenced by the composition (e.g., plasticizers). If the film is fragile, it makes it difficult or even impossible to apply it in the place of application, so it is important to make it more elastic by using plasticizers [18]. The deformation processes of polymer films have been routinely studied in the literature [19,20,21,22].

Active ingredients with small molecules easily pass through the buccal mucosa; however, the delivery of peptides and proteins is a difficult task because the drug must pass through 40–50 cell layers of which the thickness is approximately 500–600 μm [23,24]. One possible solution is to formulate micro- and nanoparticles and then incorporate the particles into the polymer film. Al-Nemrawi et al. therefore formulated insulin-containing nanoparticles incorporated into carboxymethylcellulose and HPMC polymer films [25]. Batista et al., in turn, prepared a chitosan film loaded with chitosan microparticles containing a bioactive peptide for buccal delivery [26]. Another option is to use a permeation enhancer excipient such as citric acid, menthol [27], salcaprozate sodium (SNAC), sodium caprate [28], and chitosan ascorbate, etc. It is known that chitosan can interact with buccal lipids, and it is proven that polymer salification with ascorbic acid produces a higher chitosan interaction with dietary lipids. According to the findings of Rossi et al., chitosan ascorbate had a better penetration-enhancing effect in buccal porcine mucosa as well as in Caco-2 cells [29].

Our aim was to produce ascorbic acid-containing chitosan films that are well applied as buccal mucoadhesive films. Therefore, we also aimed to determine the size of the free volumes as well as the structural, fine structural, and physical properties of the films, in order to select the composition to be further developed based on this knowledge. A further aim was to test the suitability of the optimized composition with different types of active agents. In our work, we chose an acidic component for the dissolution of chitosan, which, according to literature data, forms chitosan ascorbate with chitosan, which also has permeation-enhancing properties [29,30].

## 2. Materials and Methods

### 2.1. Materials

The polymer on which the film was based was Chitosan 80/1000 (Chitoscience Chitosan, HEPPE MEDICAL CHITOSAN GmbH, Halle, Germany), with a degree of 77.6–82.5% deacetylation and apparent viscosity of 751–1250 mPas (1% in 1% acetic acid, 20 °C). Ascorbic acid (Ph. Eur.) was used in the film as an excipient to provide acidic pH and permeation enhancement. Glycerol (Ph. Eur.) was used as a plasticizer. Acetic acid (min. 99.8%, Sigma-Aldrich, Darmstadt, Germany) provided the appropriate acidic environment for dissolution during the preparation of the reference films. Distilled water was present in the compositions as a dissolving medium. Mucin (Carl Roth GmbH + Co. KG, Karlsruhe, Germany) (10 *w/w*%) dispersion was used in the in vitro mucoadhesion test. It was isolated from the porcine stomach lining (data from the producer). Diiodomethane was used in the surface free energy measurement. Diclofenac sodium (diclofenac Na) and cetirizine dihydrochloride (cetirizine HCl) (Ph. Eur. 8.) were applied as an active ingredient in the polymer films. Phosphate buffer pH 6.8 was used for dissolution testing.

### 2.2. Methods

#### 2.2.1. Preparation of Samples

The solvent casting method [7,8,26,31] was used for the preparation of the films on the Teflon surface. This procedure is generally known as a simple way of formulating buccal films. In the case of films containing ascorbic acid, the calculated amount of the polymer and ascorbic acid was dissolved in distilled water with a motor stirrer (VELP Scientifica, Usmate, Italy) and a propeller stirrer, with continuous stirring, and glycerol was added during the process. In the case of the acetic acid film, first the appropriate dilution of the concentrated acetic acid was performed, and the measured polymer was dissolved in the 2% acetic acid solution. In the next step, glycerol was mixed with the acetic acid solution. The compositions of the prepared films are summarized in Table 1. Each film contained the same amount of 2 *w/w*% chitosan, and glycerol was also used uniformly in all films at 1 *w/w*%. Ascorbic acid was used as an excipient in a concentration range of 2–5 *w/w*%. Ten grams of the above solution was cast into rubber rings on a smooth surface, dried at a temperature of 24.5 ± 0.5 °C, and the films were cut from the rings. Based on our results, the Film 2 composition was selected as the optimal composition, so 2 different types of model active agents, cetirizine HCl and diclofenac Na, were incorporated. As a first step, the active agent was dissolved in distilled water and then added to the polymer solution.

#### 2.2.2. Breaking Strength

The breaking strength and deformation behavior of the films were measured with a computer-controlled Texture Analyzer developed at our Institute. This tool is equipped with a specific holder (20 mm in diameter) and a hemispherical indent of 1.13 mm^2^. The sensor contains UNICELL force-measuring equipment. The load cell is attached to the DAQ box, which is based on the Silicon Laboratories C8051F124 microcontroller development kit. The range of measurement was 0–200 N, the stamping speed was 20 mm/min, and the frequency of the sampling was 50 Hz. The deformation curves can be registered, and the ultimate deformation force can be calculated as the maximum value of the force–time curve. The test was performed ten times (*n* = 10) for each combination of films. The standard deviations and means were calculated. The breaking strength was calculated with a contact area of 1.13 mm^2^ according to the following formula: σ = F/A.

#### 2.2.3. In vitro Mucoadhesion Test

The breaking strength tester described in detail above was improved for the direct measurement of the adhesion force [32].

The algorithm of the mucoadhesion force measurement performs the following operations: 1. At the start of the measurement process, the pressure jowl moves downwards and presses against the polymer film until the predetermined pressure is reached (static pressure). 2. It holds its position until the desired timeout (holding time). 3. The pressure jowl will then start to move up, until the user stops the measurement process (dynamic pressure).

Double-sided adhesive tape was attached to the surface of the sample holder and the polymer film was attached to the other side of the adhesive tape. A stainless-steel holder was fixed to the lower part of the tester and 40 µL of freshly prepared mucin dispersion (10 *w/w*%) was spread on it. The rod-like sample holder was moved downwards and pressed against the lower disc covered with mucin for 50 s with a force of 50 ± 0.1 N. This steady-state part can be observed in the force–time curve. The specimen holder was then moved upwards at a stamping speed of 20 mm/min and the force was reduced until the specimen started to separate from the mucin, which can be seen as a well-defined peak in the force–time curve. The peak maximum shows the mucoadhesion force. The measurement was carried out six times (*n* = 6) and means and standard deviations were calculated.

#### 2.2.4. X-ray Powder Diffractometry (XRPD)

X-ray powder diffractometry (XRPD) uses X-rays to study and quantify the structure of films. XRPD analysis was performed by a Bruker D8 Advance diffractometer (Bruker AXS GmbH, Karlsruhe, Germany) with Cu K λI radiation (λ = 1.5406 Å). The films were scanned at 40 kV and 40 mA from 3 deg to 40 deg 2 Θ, at a scan rate of 0.1 deg/s and a step size of 0.010 deg. The amorphous/crystalline state of the investigated polymer system and the possible recrystallization tendency due to accelerated aging were investigated.

#### 2.2.5. Positron Annihilation Lifetime Spectroscopy (PALS)

A positron is an antiparticle of an electron that has the same spin and mass as an electron and the opposite charge. When a positron encounters an electron, mutual annihilation occurs, creating photons carrying information about the surroundings of the annihilating pair. The rate of annihilation is greatly influenced by the electron density in the material, so positrons can be exceptionally sensitive scanners for electron density. As local electron density is closely connected to the free volume in polymeric materials, positrons might be valuable tools for studying the free volume [33].

In polymers, a large fraction of positrons form positronium, i.e., a kind of light ‘hydrogen’ atom formed by an electron and a positron. There are two forms of positronium depending on the spin: Ortho-positronium (o-Ps) if the spin of the positron and electron is parallel, and para-positronium (p-Ps) if their spins are anti-parallel. Because of its much longer lifetime, o-Ps is used to study polymers while the short-lived p-Ps is handled as the background. In a vacuum, its lifetime is about 140 ns, which is reduced to a few ns in polymers because of the electrons in the vicinity of o-Ps, with a spin opposite to the positron. This process is the so-called pick-off annihilation. Its rate depends on the electron density greatly and, indirectly, it is governed by the sizes of free volumes around the annihilating o-Ps. This is used to study the cavity size relationships of polymer systems: The smaller the cavity the o-Ps atom enters, the sooner it encounters an electron of opposite spin during its motion [34]. For details of lifetime spectroscopy, one can refer to numerous reviews [35].

An ORTEC-PLS system (Amelek Inc., PA, USA) was used, and sodium-22 chloride (^22^NaCl) was used as the radiation source. The source activity ranged from 5 to 105 Bq at 4096 channel numbers. The resolution of the measuring system was 210 ps, while a channel had a time of ~10 ps. The detectors were BaF_2_-based scintillation probes. The rate of positronium formation was approximately 5–8% of the annihilation events.

#### 2.2.6. Atomic Force Microscopy (AFM)

The visualization of the films was carried out by atomic force microscopy (AFM) using Nanoscope IIIa type Multimode (Digital Instruments, Tonawanda, NY, USA) equipped with an “E”-type scanner. Amplitude- and height-mode images were captured at room temperature in the air using the tapping mode with a silicon tip cantilever (Bruker Corporation, Billerica, MA, USA) operated at a resonance frequency of 275–300 kHz and at a scan rate of 1.2 Hz. The images were evaluated with the Nanoscope V614r1 software (Digital Instruments, Buffalo, NY, USA).

#### 2.2.7. Scanning Electron Microscopy

Scanning electron micrographs (SEM) were taken with a Hitachi S4700 (Hitachi, Tokyo, Japan) scanning electron microscope using 10.0 kV acceleration voltage at a magnification range of 30×–1000×. A sputter coating unit (Polaron E5100, VG Microtech, Laughton, UK) was used to charge the surfaces for the SEM measurements.

#### 2.2.8. Surface Free Energy (SFE)

The contact angles of the polymer films were measured with an OCA 20 (DataPhysics Instruments GmbH) instrument, and the results were used to calculate the surface free energies (SFE). This indirect method of the evaluation of the surface free energy from wettability measurements is commonly used [36,37,38]. In Wu’s [39] method, the surface free energy is considered to be the sum of the dispersed (*γ^d^*) and polar (*γ^p^*) components. The surface free energy of solids can be determined by means of contact angle measurements with two different fluids of known polar and dispersed parts of surface tension properties (Equation (1)). The test fluids were distilled water and diiodomethane (Merck KGaA, Darmstadt, Germany). According to Ström [40], the dispersed part of the surface tension was 21.8 mN/m for water and 50.8 mN/m for diiodomethane, while the polar part of the surface tension was 51 mN/m for water and 0 mN/m for diiodomethane. Next, 0.50 g of the powders were compressed using a hydraulic press (Specac Inc, Graseby, Orpington, UK) with a dwell time of 10 s at a pressure of 200 MPa. Circle fitting was used to determine the contact angle formed on the compacts made from different samples. They can be estimated by solving an equation with two unknowns (Equation (1)):

(1) $$\left(1+cos\theta \right)\gamma_{l}=\frac{4\left(\gamma_{s}^{d}\gamma_{l}^{d}\right)}{\gamma_{s}^{d}+\gamma_{l}^{d}}+\frac{4\left(\gamma_{s}^{p}\gamma_{l}^{p}\right)}{\gamma_{s}^{p}+\gamma_{l}^{p}}$$

where *θ* is the contact angle, *γ*_s_ is the solid surface free energy, and *γ*_l_ is the liquid surface tension (superscripts refer to their polar (*γ^p^*) and dispersed part (*γ^d^*)).

#### 2.2.9. Fourier-Transform Infrared Spectroscopy (FTIR) Analysis

The Fourier-transform infrared spectra of the samples were examined using an Avatar 330 FTIR instrument (Thermo-Scientific, Waltham, MA, USA) with an attached Zn/Se horizontal attenuated total reflectance (HATR) apparatus. The films were placed on the clear crystal of the device. The wavelength range was 600–4000 cm^−1^ during the study. Spectra were obtained from 128 scans at a spectral resolution of 4 cm^−1^. CO_2_ and H_2_O were used for correction.

#### 2.2.10. Dissolution Test

For the dissolution test, 0.6 and 0.9 g film pieces (containing 38 mg and 25.5 mg of diclofenac Na and cetirizine HCl, respectively) were used. Erweka DT700 dissolution equipment with a basket tester was used during the dissolution test. The temperature was 37 °C and the mixing speed was 100 rpm. Nine hundred milliliters of phosphate buffer (pH = 6.8) were applied as dissolution medium. Aliquots were 5 mL and were analyzed in 5, 10, 15, 30, 45, and 60 min by UV-spectrophotometry (Genesys 10S UV-VIS, Thermo Fisher Scientific, USA) at λ = 276 nm (diclofenac Na) and λ = 231 nm (cetirizine HCl).

## 3. Results and Discussion

### 3.1. Breaking Strength

**Figure 1:**
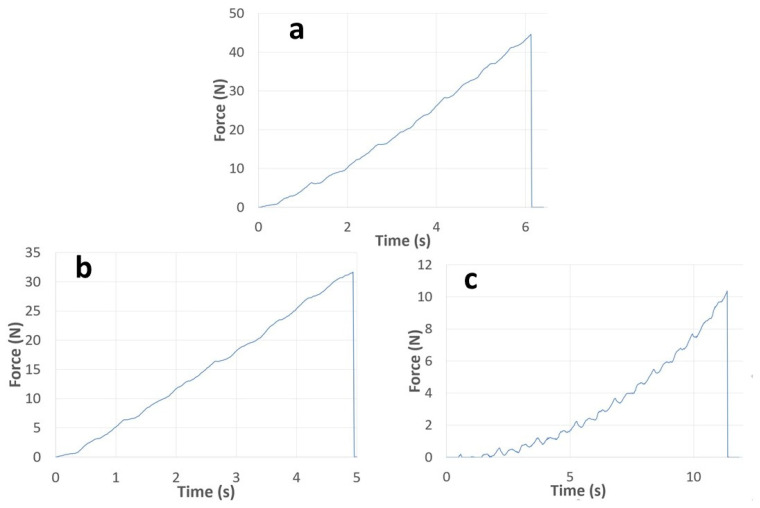
Force–time curves in the case of Ref (**a**), 2% (**b**) and 5% (**c**) concentrations of ascorbic acid.

**Figure 2:**
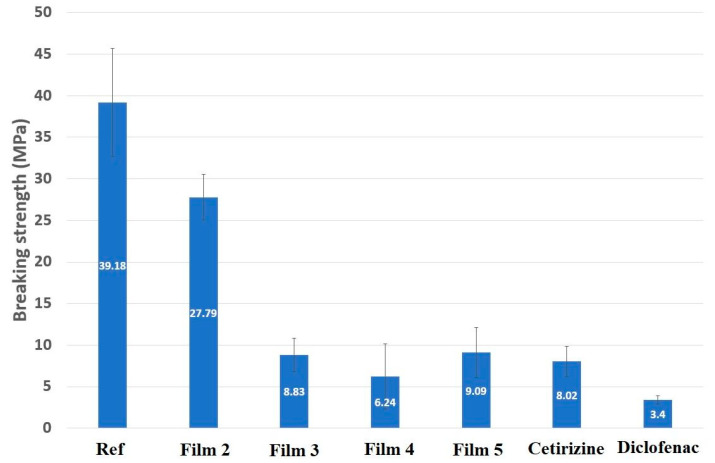
Breaking strength results in combination with acetic acid for chitosan films made with different concentrations of ascorbic acid and films containing cetirizine HCl and diclofenac Na.

In addition to the breaking strength, the deformation process of the films also provides information on the elasticity of the specimens. The breaking force of the film is the maximum value on the deformation curve, after which the measured force decreases to zero (Figure 1), and the breaking strength was calculated from this value. It can be seen that the highest value was found for the film made with acetic acid (Ref), 39.18 MPa, which can be explained by the fact that during the formation of chitosan acetate, the excess acetic acid easily evaporates from the system and thus does not affect the breaking strength (Figure 2). It can also be seen that in the case of a film containing 2% ascorbic acid (Film 2), the breaking strength is significantly lower, which is explained by the formation of chitosan ascorbate, which resulted in a different chemical structure and therefore has different properties. It can be seen that the 2% ascorbic acid sample has a higher breaking strength than the other ascorbic acid films. The molar ratio of deacetylated chitosan monomers to ascorbic acid, which reacts in a 1:1 stoichiometric ratio to form chitosan ascorbate, was determined by averaging the range of deacetylation levels specified by the manufacturer (80%). In this case, a molar ratio of 1.57:1 (ascorbic acid:chitosan) was obtained, which may be closer to a stoichiometric ratio of 1:1 if the degree of deacetylation exceeds 80%. For a film containing 3% ascorbic acid (Film 3), this ratio is 2.36:1 (ascorbic acid:chitosan), which is already much higher than the stoichiometric ratio. This means that in this case, a larger amount of ascorbic acid remains in excess, which significantly reduces the breaking strength of the film. As the concentration of ascorbic acid is increased, the amount of free ascorbic acid increases, whereby at 4% (Film 4) it is 3.14:1 and at 5% (Film 5) the molar ratio is 3.9:1 (ascorbic acid: chitosan), but in this range, the value of the breaking strength is not further reduced. The breaking strength of drug-containing films was also studied, and it was found that both active agents reduced the value of the breaking strength, but this did not decrease below the applicability. The cationic drug, cetirizine HCl, reduced the breaking strength by nearly 70% and the anionic diclofenac Na by nearly 90%. It can be seen that the cetirizine film was approximately twice as high as the diclofenac film, which may be due to the fact that cetirizine HCl can be incorporated into the film structure because it is a cationic active agent that is highly soluble in acidic environments and thus can be incorporated into the polymer chain during production. However, in the case of diclofenac Na, an anionic active agent, the drug was precipitated in an acidic environment during production, leaving small diclofenac Na particles free in the film, which reduced the breaking strength by less than half.

Figure 1 shows the deformation curves of the samples containing 2 and 5% ascorbic acid, where it can be seen that the curve has even more significant curvature at the 5% concentration than at 2% and in the Ref film, where the course of the curve is already close to linear. It can be seen that ascorbic acid at a concentration of 2% does not affect the course of the deformation curve; however, at a concentration of 5%, curvature can already be observed, suggesting that ascorbic acid at this concentration can already function as a plasticizer in the film.

### 3.2. Mucoadhesion Force

**Figure 3:**
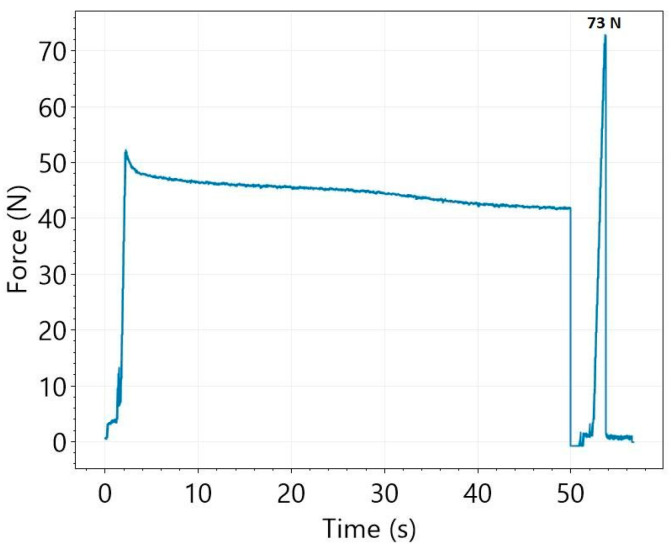
Process of measurement of mucoadhesion force (Ref).

The total measurement process of the force of adhesion is illustrated in Figure 3. According to the mucoadhesion force measurement algorithm, the resulting curve can be divided into three phases. First, the pressure jowl pushes the film with 50 N. The almost horizontal part of the force–time curve relates to the second part of the measurement procedure (holding time is 50 s). At the end of the holding time (third phase), the movement of the pressure jowl is reversed and it starts to pull upwards on the film. In this period, the peak of the force–time curve relates to the force of adhesion.

**Figure 4:**
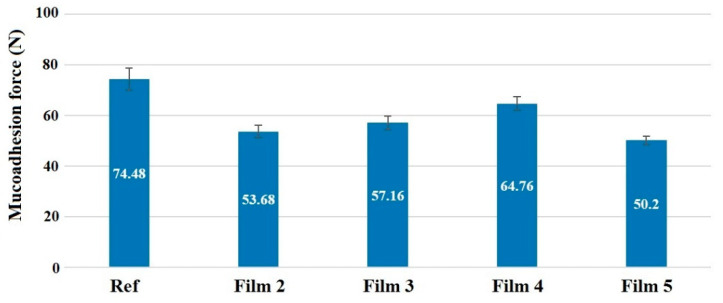
Mucoadhesion force of the films prepared with acetic acid (Ref) and different concentrations of ascorbic acid.

**Figure 5:**
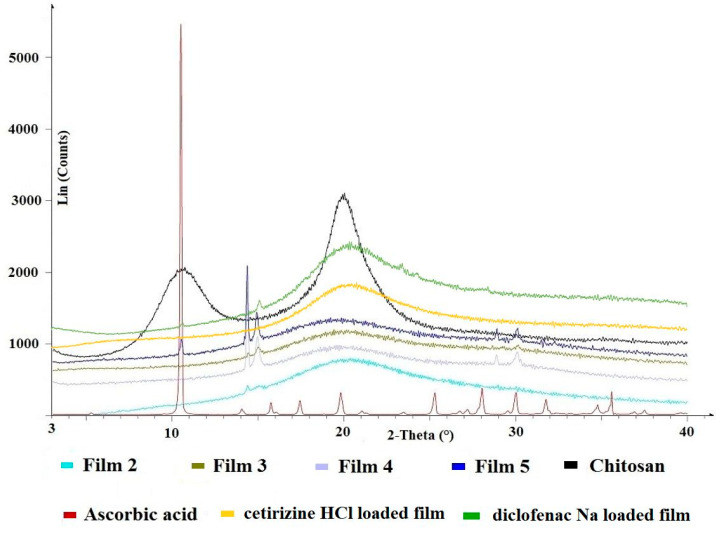
The XRPD results of the films.

Mucoadhesion force is one of the most important parameters for applicability. In order for the buccal polymer film to adhere to the mucosa, it is essential to develop an adequate degree of mucoadhesion, which can be monitored by measuring the mucoadhesive force. After the measurements, the mucoadhesion force of the different samples becomes comparable, as illustrated in Figure 4. It can be seen that the highest value was measured for the film made with acetic acid (74.48 N), and the values were much lower for all films made with ascorbic acid, so it can be concluded that ascorbic acid reduces mucoadhesion. When comparing the mucoadhesion force of samples prepared with different ascorbic acid concentrations, it can be seen that it shows a slight increase from the 2 to 4% concentration; however, the smallest mucoadhesive force (50.2 N) was measured at the 5% concentration. This can be explained by the fact that in this case, the concentration of ascorbic acid is already so high that it crystallizes freely next to the polymer, which greatly reduces the development of mucoadhesion. The functional groups of some of the mucins are attached to ascorbic acid instead of the polymer chains. The presence of free ascorbic acid is also supported by the XRPD results, and at this concentration, the characteristic peak of ascorbic acid appears first (Figure 5), therefore the application of a 2% ascorbic acid concentration can be recommended. The mucoadhesive force of cetirizine HCl- and diclofenac Na-containing films was also studied, and it was found that at the concentrations we used, the active ingredients resulted in a significant reduction in mucoadhesive force. However, they are still considered suitable for applicability. A value of 5.11 N was obtained for diclofenac Na and 8.12 N for cetirizine HCl. It can be seen that a significantly higher value was obtained for cetirizine HCl, which can be explained by the fact that cetirizine HCl could be incorporated into the polymer structure in the acidic environment, while diclofenac Na precipitated, resulting in small diclofenac Na particles in the film. As a result, the mucoadhesive force is also lower. 

### 3.3. Results of XRPD

The chitosan amorphous material, as seen on the powder X-ray spectrum, has a widening peak with relatively low intensity. In contrast, ascorbic acid has a characteristic peak with high intensity, of which the spectrum is transformed at 0.05 in the figure to show fewer characteristic peaks with sufficient intensity. If we look at the spectra of the films, it can be seen that the most characteristic peak of ascorbic acid appears in the film containing 5% ascorbic acid, but new peaks proportional to the concentration also appear (Figure 5). The literature describes a reaction whereby ascorbic acid catalyzes the hydrolysis of chitosan by radical conversion in the presence of O_2_ to form chitosan ascorbate. Ascorbic acid is as well considered to be an acid that can dissolve chitosan directly in water and concurrently form water-soluble chitosan ascorbate [37].

This may also explain the mucoadhesion measurements, namely that the 5% film composition had the lowest adhesion strength, as excess free ascorbic acid may prevent the mucin-polymer chains from diffusing into each other, so it may not result in such a strong adhesion strength, and this may cause weaker in vitro mucoadhesion (Figure 4).

It is well known from the literature that both active agents are crystalline, with many characteristic peaks on X-ray diffractograms [41,42]. In the case of drug-containing films, it can be seen that the active agents are present in an amorphous form in the polymer film. However, smaller peaks also appear for diclofenac Na, suggesting partial recrystallization.

### 3.4. Free Volumes of the Films

**Figure 6:**
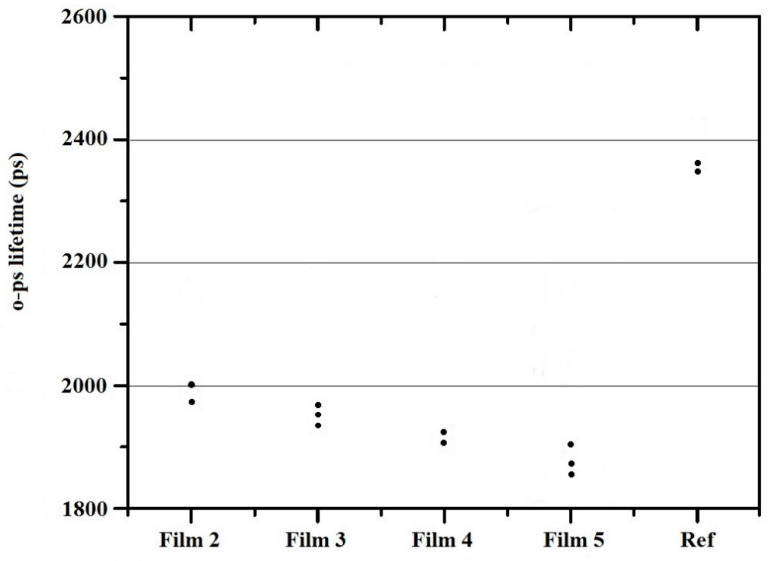
The results of PALS measurements.

It can be seen that for citric acid films, we obtained values between 1800 and 2000 ps in every case. Within this range, the o-ps lifetime decreases proportionally with an increasing ascorbic acid concentration (Figure 6). The size of the free volumes (indicated by positronium) is definitely decreasing, as the shorter positronium lifetime indicates a smaller free volume. Ascorbic acid either fills the space between the polymer chains or moves the chains closer together leaving less space for o-Ps. In any case, it is clear that the presence of ascorbic acid plays a key role in the resulting structure. The free volume of samples containing acetic acid is much larger than any ascorbic acid state (2400 ps). Acetic acid essentially completely evaporates during preparation and drying, i.e., in this case, only the chitosan-acetate remains in the structure of the films. It can be seen that increasing the concentration of ascorbic acid does not favor the size of the free volumes, which can later ensure the location of the active ingredient, therefore it can be concluded that, on the basis of o-Ps lifetime measurements, the application of a low (2%) ascorbic acid concentration is optimal for production.

### 3.5. Morphology of the Films

**Figure 7:**
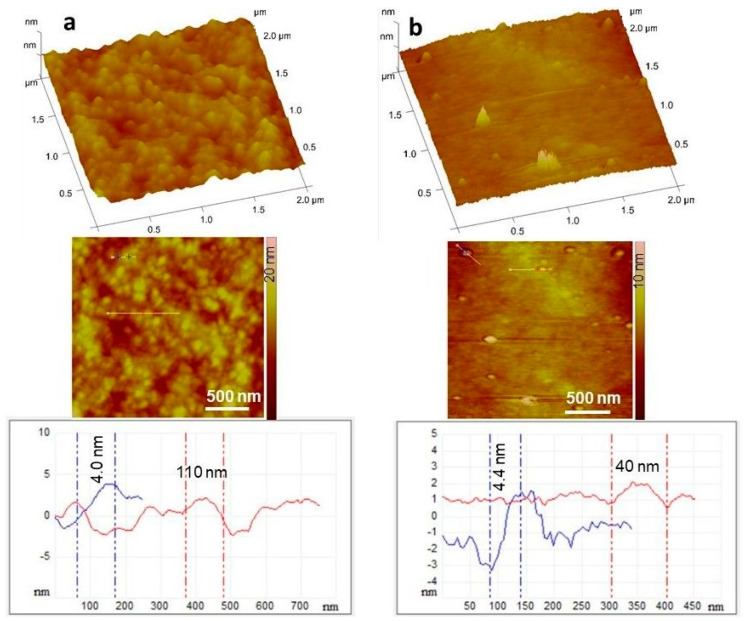
3D representation, amplitude AFM images of Ref (**a**) and Film 5 (**b**) surfaces with the section analysis curves shown along the indicated white, blue, and red lines. In the composite, the ascorbic acid content was 5% (Film 5).

The morphology of the films was visualized by the AFM technique in the amplitude and height profile representation as well as 3D and section analysis profiles. The images in Figure 7 clearly show the fine structure of the chitosan layer surface both in the absence and presence of ascorbic acid. Ascorbic acid crystal formation can be observed in the case of Film 5; however, the surface of the samples is very smooth as indicated from the section analysis profiles, with the presence of ascorbic acid crystals around 4.4 nm in height. In this case, the composition differed significantly from the stoichiometric chitosan:ascorbic acid molar ratio, so the excess ascorbic acid could crystallize freely, which was also confirmed during the XRPD analysis, as X-ray peaks characteristic of ascorbic acid appeared (Figure 5). It can also be seen that the environment of the ascorbic acid crystals is very smooth, suggesting that chitosan ascorbate is able to form a more uniform and smoother polymer film than chitosan acetate.

**Figure 8:**
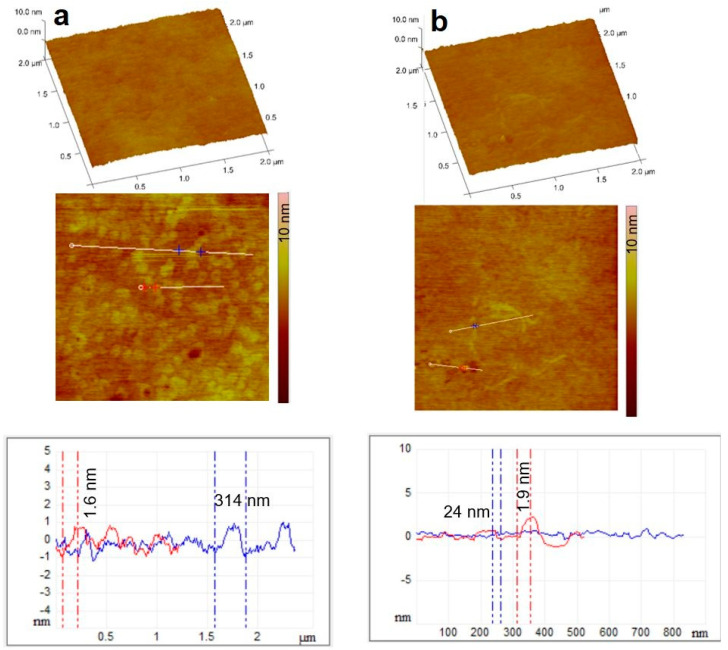
3D representation, amplitude AFM images of film containing cetirizine HCl (**a**) and diclofenac Na (**b**) surfaces with the section analysis curves shown along the indicated white, blue, and red lines. In the films, the ascorbic acid content was 2%.

The fine structure of films containing active agents was also studied with AFM. There are no significant differences in the morphology of the different drug-containing films. The surface of the cetirizine HCl-containing film is very similar to that of the reference film, which is a uniform homogeneous film containing only chitosan. Diclofenac does not show any major surface roughness either, because the diclofenac Na particles formed during the formulation are situated at the bottom of the polymer film (Figure 8).

### 3.6. Morphology of the Films

**Figure 9:**
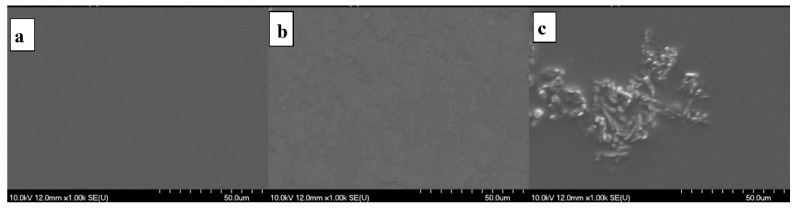
Scanning electron micrographs of chitosan ascorbate films at 1000× magnification (API-free film (Film 2) (**a**), cetirizine HCl-loaded film, (**b**) and diclofenac Na-loaded film (**c**)).

The surface of the films can be aptly studied through SEM pictures [43]. The texture of the films still seemed to be uniformly smooth for both API-free and API-containing formulations. Nevertheless, considerable differences were observed at 1000× magnification (Figure 9). High magnifications revealed that cetirizine HCl-loaded films have considerably higher surface roughness than that of the API-free film. A possible explanation of this phenomenon may be that the cationic API is embedded into the texture of the acidic film in molecularly dispersed or amorphous forms. In contrast, the anionic diclofenac Na precipitated in the acidic environment, and the precipitated API nano- or microcrystals embedded into the film are visible in Figure 9.

### 3.7. Results of SFE

From the measured contact angle results, the value of *γ^tot^* and its dispersive (*γ^d^*) and polar (*γ^p^*) components were calculated based on the Wu method. The SFEs and the polar and dispersed components of the ingredients and the films were calculated from the measured contact angle (Table 2). The dispersed part is from the London dispersion force, while the polar part is from the dipol–dipol interaction, induction force, and H-bonds. It can be seen that as the ascorbic acid concentration increases, the polar component of SPF decreases due to dipole–dipole interactions, because in this case, the chemical bond is formed through an –OH group of ascorbic acid and an –NH_2_ group of chitosan during the formation of chitosan ascorbate. It can also be seen that ascorbic acid significantly increases the surface free energy and its polar part compared to films made with acetic acid. This is explained by the fact that acetic acid evaporates from the system while ascorbic acid remains in the film, either as chitosan ascorbate or in a high amount in the form of ascorbic acid. The ascorbic acid molecule contains 4 –OH groups, so free –OH groups remain even after the formation of chitosan ascorbate.

### 3.8. Results of FTIR

**Figure 10:**
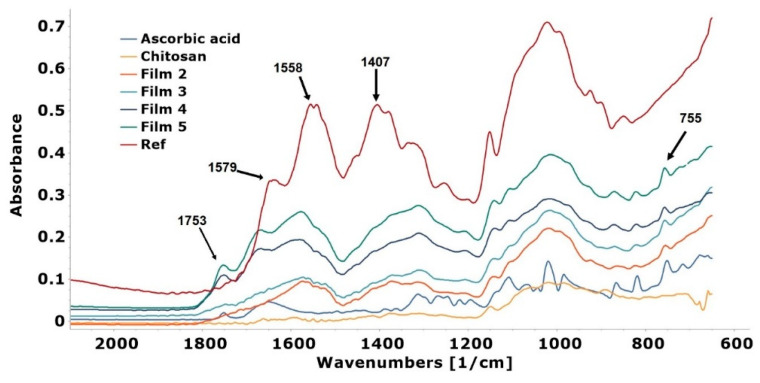
FTIR spectra of chitosan, ascorbic acid, and the films containing ascorbic acid.

In the FTIR spectra, the main difference is seen between the chitosan and Ref film: A distinctive band disappears at 1600 cm^−1^, and new peaks can be identified at 1558 and 1407 cm^−1^ (Figure 10), which, according to the literature data, indicates that chitosan acetate can be formed [44]. The appearing peaks can be attributed to the −NH_3_^+^ bending vibration and −COO− symmetric tensile vibration resulting from the electrostatic interaction. In the spectra of ascorbic acid and films containing ascorbic acid, a stretching vibration peak at 1754 cm^−1^ represents the intramolecular hydrogen bonds of the lactone carbonyl group. Based on the spectra and literature data in these films, it can be concluded that chitosan ascorbate may have been formed [45]. It can be observed that the aforementioned characteristic peak of ascorbic acid was only observed at 1753 cm^−1^ for films with 4 and 5% ascorbic acid (Film 4 and Film 5), while at lower ascorbic acid concentrations (Film 2 and Film 3), these peaks disappear. This suggests that in these cases, the total amount of ascorbic acid was converted to chitosan ascorbate. In addition, the overlapping peak of the C=C stretching vibration of chitosan ascorbate and the bending vibration of chitosan −NH_3_^+^ is observed at about 1579 cm^−1^, and the weak peaks at 755 cm^−1^ are attributed to the C=C bending vibration of chitosan ascorbate.

**Figure 11:**
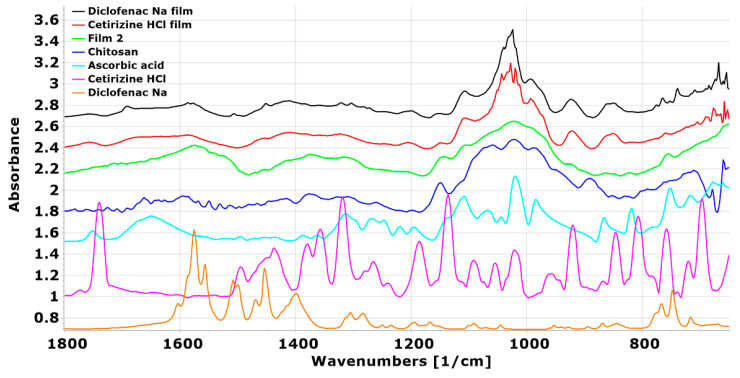
FTIR spectra of chitosan, ascorbic acid, and the films containing active agents (cetirizine HCl, diclofenac Na).

In the spectra of the drug-loaded films, it can be seen that the peaks for films containing diclofenac Na appear at the same wavenumber as in the spectrum of the pure drug (Figure 11), suggesting that diclofenac Na is indeed present in the film in the form of small free particles and is not built into the polymer film structure. However, in the case of films containing cetirizine HCl, shifts can be observed, suggesting that a stronger interaction between the active agent and the polymer may have occurred and may be incorporated into the polymer structure.

### 3.9. Results of Dissolution Test

**Figure 12:**
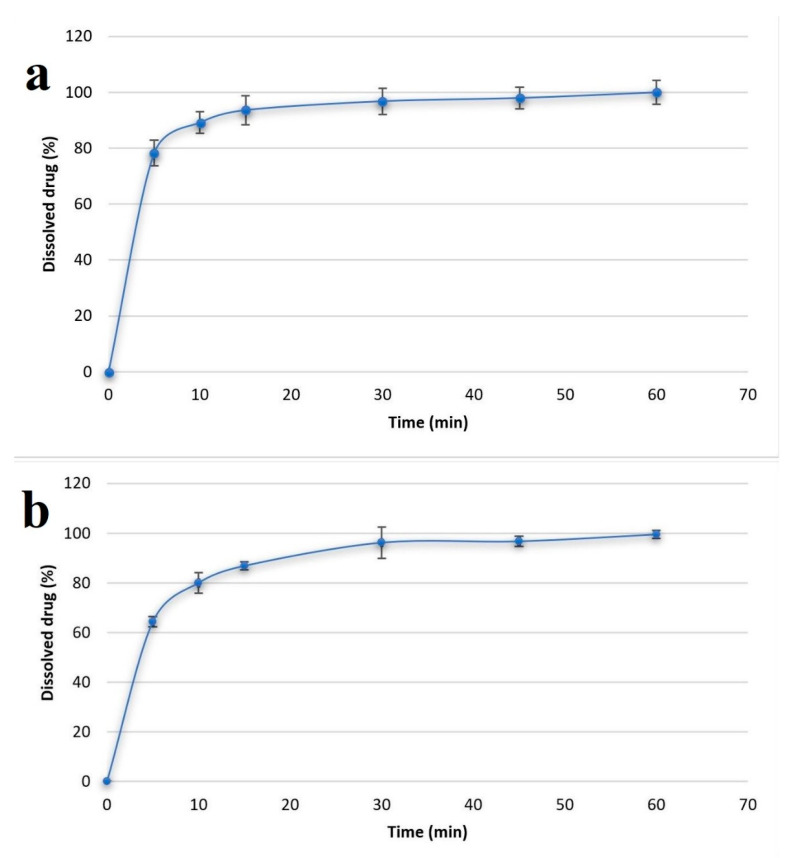
The dissolution curves in the case of diclofenac Na (**a**) and cetirizine HCl-loaded film (**b**).

During the optimization of the ascorbic acid concentration, it was found that the use of a 2% ascorbic acid concentration was optimal, so this composition was tested with two different types of model active agents. One was diclofenac Na and the other was cetirizine HCl. In both cases, the kinetics of dissolution were also studied, the results of which are shown in Figure 12. In both cases, a saturation curve was obtained, but minor differences were observed. In the case of diclofenac Na, a faster release is initially observed, presumably due to the fact that diclofenac Na is an anionic drug and precipitates in the acidic medium during formulation, so the drug can be present in its own crystalline form, as evidenced by X-ray diffractogram (Figure 5). In the case of cetirizine HCl, which remained a completely transparent solution during the formulation of the cationic drug, it is likely that the drug may be incorporated into the polymer chain, leading to an initially slower release compared to diclofenac Na. For both drugs, the total amount of the active agent could be released from the polymer film in 60 min.

## 4. Conclusions

In summary, ascorbic acid-containing chitosan films were produced as buccal drug delivery systems by casting technology. Chitosan ascorbate was formed in the ascorbic acid-containing polymer solutions used for the films, which was confirmed by FTIR spectra in the dry films. We studied the breaking strength of the films and the deformation curve obtained during the study, based on which it was established that at a concentration of 5% ascorbic acid, ascorbic acid can already function as a plasticizer. The cationic drug, cetirizine HCl, reduced the breaking strength by nearly 70% and the anionic diclofenac Na by nearly 90% because the cationic drug could be incorporated into the film structure while the anionic drug was not. The size of the free volumes decreases with an increasing ascorbic acid concentration; therefore, a low concentration (2%) is recommended during the formulation, which may be optimal for the location of the active agent to be used. The fine structure and the morphology of the films were investigated by the AFM technique, and it was found that chitosan ascorbate results in a polymer film with a very uniform and smooth surface. At a concentration of 5% ascorbic acid, ascorbic acid crystals can already be observed on both AFM images and X-ray diffractograms, so this supports the need to use ascorbic acid at the lowest possible concentration, which in this case means 2% ascorbic acid. Therefore, the 2% ascorbic acid film was tested with two different types of model drugs, one cationic and one anionic. In both cases, the film releases the drug along a saturation curve, initially faster for the anionic drug and slower for the cationic drug. The difference can be explained by the fact that the cationic drug can be easily incorporated into the structure of the film, while this cannot be said of the anionic drug, which was also confirmed by the XRPD and FTIR studies. Overall, we studied chitosan films containing ascorbic acid using analytical methods rarely used in the field of pharmaceutical technology, which provided new, important information on the structure, free volume, and surface properties of the films, which may greatly contribute to the further development of films in the future. The concentration of ascorbic acid used was optimized and it was found that the use of a concentration of 2% was optimal during the formulation. Finally, the optimized film base was tested with active agents and found to be primarily recommended for the use of cationic active agents.

## Figures and Tables

**Table 1:** The composition of the solutions for film casting.

Sample	Ascorbic Acid (*w/w*%)	Chitosan (*w/w*%)	Glycerine (*w/w*%)	Acetic Acid (*w/w*%)	Active Agent (*w/w*%)
Reference (Ref)	-	2	1	2	-
Film 2	2	2	1	-	-
Film 3	3	2	1	-	-
Film 4	4	2	1	-	-
Film 5	5	2	1	-	-
Cetirizine film	2	2	1	-	0.5
Diclofenac film	2	2	1	-	0.5

**Table 2:** Total surface free energy and its polar and dispersed components.

Sample	*γ^tot^* (mN/m)	*γ^d^* (mN/m)	*γ^p^* (mN/m)
Ref	43.87	30.55	13.53
Film 2	70.32	35.35	34.98
Film 3	66.53	37.53	29.01
Film 4	60.04	31.31	28.73
Film 5	56.93	31.13	25.80

## Data Availability

Data sharing not applicable.
